# *N* ^2^-(4-Chloro­benzyl­idene)-4-nitro­benzene-1,2-diamine

**DOI:** 10.1107/S1600536810030461

**Published:** 2010-08-11

**Authors:** Abeer Mohamed Farag, Teoh Siang Guan, Hasnah Osman, Mohd Mustaqim Rosli, Hoong-Kun Fun

**Affiliations:** a School of Chemical Sciences, Universiti Sains Malaysia, 11800 USM, Penang, Malaysia; b X-ray Crystallography Unit, School of Physics, Universiti Sains Malaysia, 11800 USM, Penang, Malaysia

## Abstract

In the title compound, C_13_H_10_ClN_3_O_2_, the dihedral angle between the two benzene rings is 3.61 (6)°. In the crystal structure, mol­ecules are linked by weak inter­molecular C—H⋯O hydrogen bonds, forming layers parallel to the *bc* plane. Short inter­molecular Cl⋯Cl contacts [3.491 (1) Å] are also observed.

## Related literature

For the applications of Schiff base compounds see: Dao *et al.* (2000 ▶); Akbar Mobinikhaledi *et al.* (2009 ▶); So *et al.* (2007 ▶); Teoh *et al.* (1997 ▶). For the stability of the temperature controller used in the data collection, see: Cosier & Glazer (1986 ▶).
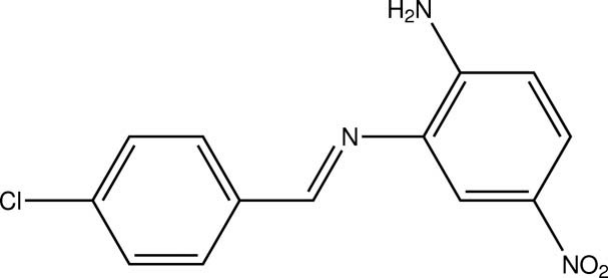


## Experimental

### 

#### Crystal data

C_13_H_10_ClN_3_O_2_
*M* *_r_* = 275.69
Monoclinic, 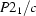

*a* = 16.969 (2) Å
*b* = 3.7852 (5) Å
*c* = 19.986 (3) Å
β = 112.373 (3)°
*V* = 1187.1 (3) Å^3^
*Z* = 4
Mo *K*α radiation
μ = 0.32 mm^−1^
*T* = 100 K
0.50 × 0.14 × 0.05 mm

#### Data collection

Bruker APEXII DUO CCD area-detector diffractometer
Absorption correction: multi-scan (*SADABS*; Bruker, 2009 ▶) *T*_min_ = 0.856, *T*_max_ = 0.984
16208 measured reflections
4441 independent reflections
3411 reflections with *I* > 2σ(*I*)
*R*_int_ = 0.048

#### Refinement

*R*[*F* ^2^ > 2σ(*F* ^2^)] = 0.045
*wR*(*F* ^2^) = 0.155
*S* = 1.07
4441 reflections
180 parameters
H atoms treated by a mixture of independent and constrained refinement
Δρ_max_ = 0.55 e Å^−3^
Δρ_min_ = −0.35 e Å^−3^

### 

Data collection: *APEX2* (Bruker, 2009 ▶); cell refinement: *SAINT* (Bruker, 2009 ▶); data reduction: *SAINT*; program(s) used to solve structure: *SHELXTL* (Sheldrick, 2008 ▶); program(s) used to refine structure: *SHELXTL*; molecular graphics: *SHELXTL*; software used to prepare material for publication: *SHELXTL* and *PLATON* (Spek, 2009 ▶).

## Supplementary Material

Crystal structure: contains datablocks global, I. DOI: 10.1107/S1600536810030461/lh5102sup1.cif

Structure factors: contains datablocks I. DOI: 10.1107/S1600536810030461/lh5102Isup2.hkl

Additional supplementary materials:  crystallographic information; 3D view; checkCIF report

## Figures and Tables

**Table 1:** Hydrogen-bond geometry (Å, °)

*D*—H⋯*A*	*D*—H	H⋯*A*	*D*⋯*A*	*D*—H⋯*A*
C7—H7*A*⋯O2^i^	0.93	2.56	3.469 (2)	165
C11—H11*A*⋯O1^ii^	0.93	2.54	3.2155 (17)	130

Symmetry codes: (i) 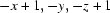; (ii) 
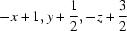
.

**Fig. 1.**
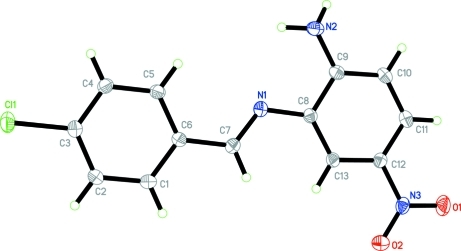
The molecular structure, showing 50% probability displacement ellipsoids and the atom-numbering scheme. Hydrogen atoms are shown as spheres of arbitrary radius.

**Fig. 2.**
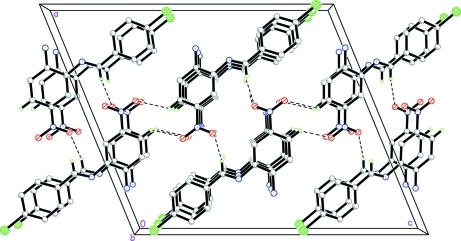
The crystal packing of (I) viewed down the b axis. Dashed lines indicate hydrogen bonds. H atoms not involved in the hydrogen bond interactions have been omitted for clarity.

## supplementary crystallographic information

### Comment

Schiff base compounds have received much attention because of their potential applications. Some of these compounds exhibit various pharmacological activities including anticancer and antibacterial properties (Dao *et al.*, 2000). Imine-type Schiff bases derived from aromatic amines and aromatic aldehydes are of growing interests because of their applications in many fields, including biological, inorganic, and analytical chemistry (Akbar Mobinikhaledi *et al.*, 2009). In another application, So *et al.* (2007) synthesized and characterized a series of Schiff base derivatives, which exhibit liquid crystal properties. Some of these Schiff bases were found to form suitable inner coordination spheres bonding to tin atom with O and N atoms as quadridentate chelates (Teoh *et al.*, 1997). Herein, we report the crystal structure of the title compound (I).

The geometrical paramters of (I), Fig.1, are within normal ranges. The dihedral angle between the two benzene rings (C1—C6) and (C8—C13) is 3.61 (6)°. The nitro group is almost co-planar with the attached C8—C13 benzene ring with dihedral angle of 3.4 (1)°.

In the crystal structure, (Fig. 2), the molecules are connected by intermolecular C7—H7A···O2^i^ and C11—H11A···O1^ii^ (see Table 1 for symmetry codes) hydrogen bonds forming layers parallel to bc plane. Short Cl1···Cl1 [3.491 (1)Å] contacts also observed in the crystal structure.

### Experimental

The title compound was synthesized by adding 4-chlorobenzaldehyde (0.562 g, 4 mol) to the solution of 4-nitrobenzene-1,2-diamine (0.306 g, 2 mol) in methanol (30 ml). The mixture was refluxed for 3 h and left stirring overnight at room temperature. The resultant solid obtained was then filtered. Yellow needle-shaped single crystals suitable for X-ray structure determination were formed after slow evaporation of solvent at room temperature.

### Refinement

The H atoms attached to N2 were located from a difference map and refined isotropically. The remaining H atoms were positioned geometrically and refined using a riding model [C–H = 0.93 Å , U_iso_(H) = 1.2U_eq_(C)].

### Crystal data

**Table:**

C_13_H_10_ClN_3_O_2_	*F*(000) = 568
*M**_r_* = 275.69	*D*_x_ = 1.543 Mg m^−^^3^
Monoclinic, *P*2_1_/*c*	Mo *K*α radiation, λ = 0.71073 Å
Hall symbol: -P 2ybc	Cell parameters from 3136 reflections
*a* = 16.969 (2) Å	θ = 3.0–32.8°
*b* = 3.7852 (5) Å	µ = 0.32 mm^−^^1^
*c* = 19.986 (3) Å	*T* = 100 K
β = 112.373 (3)°	Needle, yellow
*V* = 1187.1 (3) Å^3^	0.50 × 0.14 × 0.05 mm
*Z* = 4	

### Data collection

**Table:**

Bruker APEXII DUO CCD area-detector diffractometer	4441 independent reflections
Radiation source: fine-focus sealed tube	3411 reflections with *I* > 2σ(*I*)
graphite	*R*_int_ = 0.048
φ and ω scans	θ_max_ = 33.0°, θ_min_ = 1.3°
Absorption correction: multi-scan (*SADABS*; Bruker, 2009)	*h* = −25→25
*T*_min_ = 0.856, *T*_max_ = 0.984	*k* = −5→5
16208 measured reflections	*l* = −30→30

### Refinement

**Table:**

Refinement on *F*^2^	Primary atom site location: structure-invariant direct methods
Least-squares matrix: full	Secondary atom site location: difference Fourier map
*R*[*F*^2^ > 2σ(*F*^2^)] = 0.045	Hydrogen site location: inferred from neighbouring sites
*wR*(*F*^2^) = 0.155	H atoms treated by a mixture of independent and constrained refinement
*S* = 1.07	*w* = 1/[σ^2^(*F*_o_^2^) + (0.0908*P*)^2^] where *P* = (*F*_o_^2^ + 2*F*_c_^2^)/3
4441 reflections	(Δ/σ)_max_ < 0.001
180 parameters	Δρ_max_ = 0.55 e Å^−^^3^
0 restraints	Δρ_min_ = −0.35 e Å^−^^3^

### Special details

**Table:**

Experimental. The crystal was placed in the cold stream of an Oxford Cyrosystems Cobra open-flow nitrogen cryostat (Cosier & Glazer, 1986) operating at 100.0 (1) K.
Geometry. All esds (except the esd in the dihedral angle between two l.s. planes) are estimated using the full covariance matrix. The cell esds are taken into account individually in the estimation of esds in distances, angles and torsion angles; correlations between esds in cell parameters are only used when they are defined by crystal symmetry. An approximate (isotropic) treatment of cell esds is used for estimating esds involving l.s. planes.
Refinement. Refinement of F^2^ against ALL reflections. The weighted R-factor wR and goodness of fit S are based on F^2^, conventional R-factors R are based on F, with F set to zero for negative F^2^. The threshold expression of F^2^ > 2sigma(F^2^) is used only for calculating R-factors(gt) etc. and is not relevant to the choice of reflections for refinement. R-factors based on F^2^ are statistically about twice as large as those based on F, and R- factors based on ALL data will be even larger.

### Fractional atomic coordinates and isotropic or equivalent isotropic displacement parameters (Å^2^)

**Table:**

	*x*	*y*	*z*	*U*_iso_*/*U*_eq_	
Cl1	0.02638 (2)	0.24212 (11)	0.079898 (17)	0.02738 (12)	
O1	0.57994 (6)	0.4164 (4)	0.69359 (5)	0.0316 (3)	
O2	0.55664 (7)	0.1900 (3)	0.58829 (6)	0.0266 (2)	
N1	0.25047 (6)	0.5157 (3)	0.43505 (6)	0.0179 (2)	
N2	0.19596 (8)	0.8229 (4)	0.53077 (7)	0.0238 (3)	
N3	0.53280 (7)	0.3567 (3)	0.63014 (6)	0.0202 (2)	
C1	0.23385 (8)	0.1616 (4)	0.26105 (7)	0.0183 (2)	
H1A	0.2887	0.0703	0.2750	0.022*	
C2	0.17826 (8)	0.1407 (4)	0.18905 (7)	0.0180 (2)	
H2A	0.1956	0.0378	0.1546	0.022*	
C3	0.09674 (8)	0.2754 (3)	0.16947 (7)	0.0173 (2)	
C4	0.06938 (8)	0.4328 (4)	0.21968 (7)	0.0181 (2)	
H4A	0.0144	0.5231	0.2055	0.022*	
C5	0.12512 (8)	0.4529 (4)	0.29099 (7)	0.0174 (2)	
H5A	0.1074	0.5568	0.3251	0.021*	
C6	0.20807 (8)	0.3188 (3)	0.31270 (7)	0.0153 (2)	
C7	0.26832 (8)	0.3427 (4)	0.38775 (7)	0.0177 (2)	
H7A	0.3208	0.2296	0.4014	0.021*	
C8	0.30997 (7)	0.5466 (3)	0.50687 (6)	0.0159 (2)	
C9	0.27913 (8)	0.7203 (3)	0.55518 (7)	0.0174 (2)	
C10	0.33401 (9)	0.7706 (4)	0.62766 (7)	0.0190 (3)	
H10A	0.3139	0.8835	0.6593	0.023*	
C11	0.41718 (8)	0.6551 (4)	0.65241 (7)	0.0191 (2)	
H11A	0.4534	0.6898	0.7004	0.023*	
C12	0.44589 (7)	0.4852 (3)	0.60417 (6)	0.0164 (2)	
C13	0.39396 (7)	0.4290 (3)	0.53246 (6)	0.0161 (2)	
H13A	0.4150	0.3137	0.5016	0.019*	
H1N2	0.1659 (15)	0.846 (7)	0.4818 (13)	0.049 (7)*	
H2N2	0.1810 (14)	0.980 (6)	0.5534 (11)	0.041 (6)*	

### Atomic displacement parameters (Å^2^)

**Table:**

	*U*^11^	*U*^22^	*U*^33^	*U*^12^	*U*^13^	*U*^23^
Cl1	0.02798 (19)	0.0325 (2)	0.01613 (17)	0.00207 (13)	0.00220 (13)	−0.00210 (12)
O1	0.0213 (5)	0.0505 (7)	0.0174 (5)	0.0004 (5)	0.0010 (4)	0.0019 (5)
O2	0.0217 (5)	0.0345 (6)	0.0253 (5)	0.0060 (4)	0.0109 (4)	0.0009 (4)
N1	0.0172 (4)	0.0197 (5)	0.0156 (5)	−0.0002 (4)	0.0048 (4)	−0.0007 (4)
N2	0.0188 (5)	0.0303 (7)	0.0235 (6)	0.0027 (5)	0.0094 (4)	−0.0043 (5)
N3	0.0168 (5)	0.0256 (6)	0.0175 (5)	−0.0017 (4)	0.0058 (4)	0.0050 (4)
C1	0.0172 (5)	0.0182 (6)	0.0207 (6)	0.0017 (4)	0.0084 (4)	−0.0001 (5)
C2	0.0198 (5)	0.0182 (6)	0.0177 (5)	0.0005 (5)	0.0089 (4)	−0.0017 (5)
C3	0.0193 (5)	0.0159 (6)	0.0162 (5)	−0.0017 (4)	0.0062 (4)	0.0008 (4)
C4	0.0156 (5)	0.0186 (6)	0.0192 (5)	0.0013 (4)	0.0057 (4)	0.0000 (5)
C5	0.0170 (5)	0.0181 (6)	0.0184 (5)	0.0003 (4)	0.0080 (4)	−0.0018 (5)
C6	0.0163 (5)	0.0138 (5)	0.0166 (5)	−0.0013 (4)	0.0071 (4)	−0.0011 (4)
C7	0.0155 (5)	0.0184 (6)	0.0178 (5)	0.0000 (4)	0.0048 (4)	0.0001 (5)
C8	0.0166 (5)	0.0161 (5)	0.0153 (5)	−0.0014 (4)	0.0065 (4)	−0.0001 (4)
C9	0.0179 (5)	0.0167 (6)	0.0194 (6)	−0.0019 (4)	0.0092 (4)	−0.0006 (4)
C10	0.0223 (6)	0.0198 (6)	0.0171 (5)	−0.0029 (5)	0.0100 (5)	−0.0030 (5)
C11	0.0221 (6)	0.0201 (6)	0.0157 (5)	−0.0046 (5)	0.0080 (4)	−0.0012 (5)
C12	0.0149 (5)	0.0181 (6)	0.0164 (5)	−0.0021 (4)	0.0060 (4)	0.0024 (4)
C13	0.0175 (5)	0.0168 (5)	0.0147 (5)	−0.0002 (4)	0.0070 (4)	0.0012 (4)

### Geometric parameters (Å, °)

**Table:**

Cl1—C3	1.7375 (13)	C4—C5	1.3805 (17)
O1—N3	1.2357 (15)	C4—H4A	0.9300
O2—N3	1.2325 (16)	C5—C6	1.4009 (17)
N1—C7	1.2775 (17)	C5—H5A	0.9300
N1—C8	1.4107 (15)	C6—C7	1.4615 (17)
N2—C9	1.3624 (18)	C7—H7A	0.9300
N2—H1N2	0.92 (2)	C8—C13	1.3914 (17)
N2—H2N2	0.84 (2)	C8—C9	1.4224 (18)
N3—C12	1.4486 (16)	C9—C10	1.4054 (19)
C1—C2	1.3906 (18)	C10—C11	1.3770 (19)
C1—C6	1.3980 (17)	C10—H10A	0.9300
C1—H1A	0.9300	C11—C12	1.3919 (18)
C2—C3	1.3837 (18)	C11—H11A	0.9300
C2—H2A	0.9300	C12—C13	1.3838 (17)
C3—C4	1.3900 (18)	C13—H13A	0.9300
			
C7—N1—C8	121.07 (11)	C1—C6—C7	119.40 (11)
C9—N2—H1N2	119.2 (14)	C5—C6—C7	121.55 (11)
C9—N2—H2N2	119.4 (15)	N1—C7—C6	121.40 (11)
H1N2—N2—H2N2	110 (2)	N1—C7—H7A	119.3
O2—N3—O1	122.60 (12)	C6—C7—H7A	119.3
O2—N3—C12	118.76 (11)	C13—C8—N1	125.71 (11)
O1—N3—C12	118.63 (12)	C13—C8—C9	119.23 (11)
C2—C1—C6	120.58 (11)	N1—C8—C9	115.05 (11)
C2—C1—H1A	119.7	N2—C9—C10	121.35 (12)
C6—C1—H1A	119.7	N2—C9—C8	119.16 (12)
C3—C2—C1	118.88 (12)	C10—C9—C8	119.45 (11)
C3—C2—H2A	120.6	C11—C10—C9	120.91 (12)
C1—C2—H2A	120.6	C11—C10—H10A	119.5
C2—C3—C4	121.77 (12)	C9—C10—H10A	119.5
C2—C3—Cl1	119.05 (10)	C10—C11—C12	118.62 (12)
C4—C3—Cl1	119.18 (10)	C10—C11—H11A	120.7
C5—C4—C3	118.89 (11)	C12—C11—H11A	120.7
C5—C4—H4A	120.6	C13—C12—C11	122.39 (11)
C3—C4—H4A	120.6	C13—C12—N3	118.65 (11)
C4—C5—C6	120.83 (11)	C11—C12—N3	118.95 (11)
C4—C5—H5A	119.6	C12—C13—C8	119.40 (11)
C6—C5—H5A	119.6	C12—C13—H13A	120.3
C1—C6—C5	119.04 (11)	C8—C13—H13A	120.3
			
C6—C1—C2—C3	−0.5 (2)	N1—C8—C9—N2	3.61 (18)
C1—C2—C3—C4	0.5 (2)	C13—C8—C9—C10	0.17 (19)
C1—C2—C3—Cl1	−178.76 (10)	N1—C8—C9—C10	−178.77 (11)
C2—C3—C4—C5	−0.4 (2)	N2—C9—C10—C11	177.75 (13)
Cl1—C3—C4—C5	178.89 (10)	C8—C9—C10—C11	0.2 (2)
C3—C4—C5—C6	0.3 (2)	C9—C10—C11—C12	−0.3 (2)
C2—C1—C6—C5	0.4 (2)	C10—C11—C12—C13	0.1 (2)
C2—C1—C6—C7	−178.94 (12)	C10—C11—C12—N3	−178.79 (12)
C4—C5—C6—C1	−0.32 (19)	O2—N3—C12—C13	−2.73 (18)
C4—C5—C6—C7	179.05 (12)	O1—N3—C12—C13	177.81 (12)
C8—N1—C7—C6	−178.02 (11)	O2—N3—C12—C11	176.18 (13)
C1—C6—C7—N1	172.72 (13)	O1—N3—C12—C11	−3.29 (19)
C5—C6—C7—N1	−6.7 (2)	C11—C12—C13—C8	0.3 (2)
C7—N1—C8—C13	6.3 (2)	N3—C12—C13—C8	179.15 (12)
C7—N1—C8—C9	−174.84 (12)	N1—C8—C13—C12	178.42 (12)
C13—C8—C9—N2	−177.44 (13)	C9—C8—C13—C12	−0.40 (19)

### Hydrogen-bond geometry (Å, °)

**Table:**

*D*—H···*A*	*D*—H	H···*A*	*D*···*A*	*D*—H···*A*
C7—H7A···O2^i^	0.93	2.56	3.469 (2)	165.
C11—H11A···O1^ii^	0.93	2.54	3.2155 (17)	130.

Symmetry codes: (i) −*x*+1, −*y*, −*z*+1; (ii) −*x*+1, *y*+1/2, −*z*+3/2.
